# Temperament and Character Profile and Its Clinical Correlates in Male Patients with Dual Schizophrenia

**DOI:** 10.3390/jcm9061876

**Published:** 2020-06-16

**Authors:** Laura Río-Martínez, Julia E. Marquez-Arrico, Gemma Prat, Ana Adan

**Affiliations:** 1 Department of Clinical Psychology and Psychobiology, School of Psychology, University of Barcelona, Passeig de la Vall d’Hebrón 171, 08035 Barcelona, Spain; laurario@ub.edu (L.R.-M.); jmarquez@ub.edu (J.E.M.-A.); gprat@ub.edu (G.P.); 2 Institute of Neurosciences, University of Barcelona, 08035 Barcelona, Spain

**Keywords:** temperament, character, personality, substance use disorder, schizophrenia, dual schizophrenia, psychiatric symptoms, global functioning

## Abstract

Personality traits are relevant in understanding substance use disorders (SUD) and schizophrenia (SZ), but few works have also included patients with dual schizophrenia (SZ+) and personality traits. We explored personality profile in a sample of 165 male patients under treatment, using the Temperament and Character Inventory-Revised. The participants were assigned to three groups of 55 patients each, according to previous diagnosis: SUD, SZ- and SZ+ (without/with SUD). We analyzed their clinical characteristics, relating them to personality dimensions. The SUD and SZ+ groups scored higher than SZ- in Novelty/Sensation Seeking. SZ- and SZ+ presented higher Harm Avoidance and lower Persistence than the SUD group. SZ+ patients showed the lowest levels of Self-directedness, while SZ- and SZ+ had higher scores in Self-transcendence than the SUD group. Several clinical characteristics were associated with personality dimensions depending on diagnosis, and remarkably so for psychiatric symptoms in the SZ- and SZ+ groups. The three groups had a maladaptive personality profile compared to general population. Our results point to different profiles for SUD versus SZ, while both profiles appear combined in the SZ+ group, with extreme scores in some traits. Thus, considering personality endophenotypes in SZ+ could help in designing individualized interventions for this group.

## 1. Introduction

Personality can be broadly defined as the pattern of a person’s thoughts, behaviors, and feelings in different contexts throughout their life. From a dimensional perspective, some research supports the existence of a series of features that follow a normal distribution along a continuum, whose extremes would imply some vulnerability for the development of psychopathology [1]. Studying the relationships between mental disorders and personality traits, as well as between the latter and the clinical characteristics of some disorders, can contribute to generating new approaches and tools aimed at the prevention and treatment of psychopathology from an individualized perspective [2].

On the other hand, substance use disorders (SUD) constitute a public health problem given their high prevalence and consequences on individuals, their environment, and society as a whole [3]. Schizophrenia (SZ) is one of the mental disorders causing the greatest deterioration and stigma [4]. Furthermore, there is a high comorbidity between SUD and SZ [5], with prevalence rates of SUD of around 50% among patients diagnosed with SZ or other psychotic disorders [4,6]. This condition, called dual schizophrenia (SZ+), is more prevalent in men, as is the case with other profiles of dual diagnosis (DD) [7,8]. SZ+ has aroused great interest due to its severity, since these patients present a worse clinical and sociodemographic profile [9,10,11], less adherence to treatment, worse therapeutic results [5,12], an earlier onset of SZ and of the SUD [13,14,15], more suicide attempts [16] and more violent behavior [17], when compared to patients with a single diagnosis. Furthermore, treatment of SZ+ patients involves significant difficulties associated with their own characteristics, but also with those of the healthcare system [18].

Although much of the research on personality in DD has followed a categorical perspective in the analysis of relationships between personality disorders and SUD [19,20], studying personality from a dimensional perspective has become relevant in understanding entities such as SUD [21,22], SZ [23,24], and DD [25,26]. However, there are few available papers addressing personality traits in patients with SZ+. Collecting scientific evidence regarding SZ+ patients is a complex process, and sometimes the data have been obtained by extrapolation from works analyzing either SUD or SZ- patients separately [27]. Among the available personality trait models, Cloninger’s [28] stands out as a theoretically robust model based on a psychobiological perspective, and has been used in several studies with these diagnostic groups [29,30,31]. This model defines personality through individual differences in the adaptive systems that receive, process and store information. It is structured around two basic concepts: temperament and character. Temperament is characterized by those biological traits of personality with a larger genetic load, developing in earlier life phases, and remaining relatively stable through the life cycle. Character, on the other hand, is formed by those traits learned through experience, more related to social interactions and thus being less stable in comparison. In Cloninger’s model, personality is understood as the result of the interaction between temperament and character.

Furthermore, the evidence points to some personality traits acting as endophenotypes or risk factors for SUD development, the most relevant being Impulsivity [32,33] and Neuroticism [22,34], although some works point to an interaction between Impulsivity and anxious personality [35,36]. Furthermore, Novelty/Sensation Seeking has also been consistently associated with substance use [37,38], and high scores in Impulsivity and Novelty/Sensation Seeking have been found to be associated with a higher number of relapses [39,40], more craving and greater severity of addiction [41,42], more risk of suicide [43], higher rates of abandonment of treatment [44] and worse therapeutic results [42,45]. Using Cloninger’s model, SUD patients have scored lower in Self-directedness, Persistence, and Cooperativeness [38,44,46], low scores in the latter two being also associated with a greater probability of abandonment of treatment [47].

Research on personality has also highlighted the existence of possible endophenotypes for SZ, with Harm Avoidance, measured using Cloninger’s model, receiving the most attention [29,48]. Some studies have found an association between high Harm Avoidance and an increased risk of suicide in stabilized and under-treatment SZ patients [49,50]. Thus, studies focusing on personality assessment following Cloninger’s model point to a specific character and temperament profile made up of two components: the asocial component, characterized by high Harm Avoidance and low Reward Dependence; and the schizotypal component, characterized by high Self-transcendence, and low Self-directedness and Cooperativeness. This schizotypal profile has been proposed as a possible vulnerability marker for the development of SZ [29,31,51].

The scarce data published on SZ+ patients suggest that they have a character and temperament profile different from that observed in other groups with DD [25,30]. In some studies, the SZ+ group presented a profile similar to that of the SZ- group, but with higher scores in Novelty/Sensation Seeking [30,52], this trait also being associated with greater severity of addiction [9]. Moreover, increased Harm Avoidance was associated with the presence of more psychiatric symptoms in SZ+ patients [30]. Finally, the data point to the existence of a more marked profile in SZ+ patients when compared to those with SZ- or SUD, which worsens with age or time of consumption [52,53].

We consider that research on personality traits and possible behavioral endophenotypes is of special interest, since such knowledge can improve the design of strategies aimed at prevention as well as personalized interventions. For this reason, we decided to investigate the possible differences in temperament and character profiles among groups of SUD, SZ+, and SZ- patients under treatment, following Cloninger’s psychobiological model, and then compared them with the corresponding normative data. In addition, we analyzed whether personality traits are associated with some clinical characteristics of these disorders. To our knowledge, this is the first work focused on studying the temperament and character profile in these three diagnostic groups, and one of the few that also analyzes their personality profile.

## 2. Experimental Section

### 2.1. Participants

The total sample of our study consisted of 165 patients, all of them males, assigned to three groups of 55 patients each, according to their previous diagnosis. All the participants were under treatment in different public or private centers in the province of Barcelona (Catalonia). In the SUD and SZ+ groups, abstinence was verified by urinalysis in the referral centers.

The inclusion criteria were: (1) male sex (given the higher prevalence rates of the diagnoses studied for this sex); (2) aged 18 to 55; (3) under treatment and stabilized; (4) with a SUD diagnosis in initial remission for the SUD and SZ+ groups, according to Diagnostic and Statistical Manual of Mental Disorders (DSM-5) criteria [54]; (5) with a diagnosis of schizophrenia for the SZ- and SZ+ groups, according to DSM-5 criteria [54]. The exclusion criteria were: (1) presenting a disorder induced by substance use or medical illness, according to DSM-5 criteria [54]; (2) not yet stabilized; (3) presenting any physical and/or mental condition that could affect either understanding or taking the tests.

### 2.2. Procedure 

First, the reference professionals from the collaborating centers screened those patients who met our inclusion criteria. Then, we contacted each participant, provided more detailed information, and obtained their informed and signed consent. Participation in the study was voluntary, and the only compensation the participants received was an individualized return of their results. The Research Committee of the University of Barcelona approved our study (IRB00003099), which complied with the ethical principles of the Declaration of Helsinki [55]. A psychologist from our research team administered the assessment protocol in a variable number of sessions, depending on the state of each patient. The sessions included the assessment of other areas as part of a larger research project, with a total average of 4–5 sessions per patient. The research project, named “Psychobiology of dual diagnosis”, aims to assess the genetic polymorphisms, neuropsychological functioning, circadian rhythmicity, and personality traits in patients with SUD, DD, and severe mental illness. As a comorbid condition, the DD and severe mental illness groups include SZ, bipolar disorder, and major depressive disorder.

### 2.3. Measures

#### 2.3.1. Sociodemographic and Clinical Variables

For our study, we designed an *ad hoc* structured interview, in order to collect data regarding age, marital status, cohabitation, educational level, and employment situation, among others. In addition, through contact with the reference professionals in each center, we obtained information on the diagnoses, age of onset, family psychiatric history, suicide attempts, medical comorbidities, and relevant prescribed medication (the doses of antipsychotic drugs were converted to milligrams of chlorpromazine). Regarding substance use, we recorded the quantity and type of substances consumed, period of abstinence, and number of previous relapses. In addition, we administered the Structured Clinical Interview (SCID-I) for the DSM-IV [56] to confirm the diagnoses and complete the data collected. We applied the DSM-IV version of the SCID-I because, at the time of assessment, the Spanish version for the DSM-5 was not yet available. Additionally, we administered the Global Assessment of Functioning (GAF) scale [57] to assess each patient’s general functioning.

We used the Spanish version of the Positive and Negative Syndrome Scale (PANSS) [58] to assess psychotic symptoms in the SZ+ and SZ- participants. This instrument provides scores on a positive symptom scale, a negative symptom scale, and a general psychopathology scale. Severity of addiction in the SUD and SZ+ groups was assessed with the Spanish version of the Drug Abuse Screening Test (DAST-20) [59]. This instrument provides a total score ranging from 0 to 20, with five cut-off points (0 no addiction; 1–5 mild addiction; 6–10 intermediate addiction; 11–15 high addiction; 16–20 severe addiction).

#### 2.3.2. Temperament and Character Assessment 

We administered the Temperament and Character Inventory-Revised (TCI-R) [60], based on Cloninger’s personality model [28], to obtain the temperament and character profile of the participants in our study. This inventory consists of 240 items (5 of which are validity items) with a Likert-type response format ranging from 1 (false) to 5 (true), and offers direct scores and percentiles in seven dimensions. The four Temperament dimensions are Novelty Seeking (tendency to avoid routine and monotony, and to present a marked exploratory activity in the face of novelty); Harm Avoidance (tendency to experience negative affect, pessimism and behavioral inhibition); Reward Dependence (intense responses to rewards, including social rewards); and Persistence (persisting despite frustration or fatigue). The three Character dimensions are Self-directedness (ability to self-regulate and take responsibility for one’s behavior according to interests and values, as well as to set goals for oneself); Cooperativeness (adapting to the social environment, being able to put oneself in the place of others); and Self-transcendence (tendency to spirituality and magical thinking). This inventory has previously shown good psychometric properties, and in our total sample the internal consistency was adequate for all the scales, with the following Cronbach’s alpha coefficients: Novelty Seeking 0.745, Harm Avoidance 0.872, Reward Dependence 0.866, Persistence 0.893, Self-directedness 0.850, Cooperativeness 0.835, and Self-transcendence 0.825. 

### 2.4. Data Analysis

Main descriptive data (mean, standard deviation or standard errors and percentages) were obtained for all the measured variables. For the clinical and sociodemographic data, we explored possible differences among the three groups with univariate analyses of variance (ANOVA) for continuous data, and with Kruskal-Wallis tests for non-continuous or categorical data. When the variables affected only two groups (data relating to SZ or SUD diagnoses), we applied the Student´s t-test (t) if the quantitative data fulfilled the necessary conditions; otherwise, we used the Mann-Whitney U test. Chi-Square contrast was applied for categorical variables. Regarding internal consistency, we calculated Cronbach´s alpha coefficient for the seven TCI-R dimensions. 

We also performed multivariate analyses of covariance (MANCOVA), introducing the TCI-R dimensions as dependent variables, the group as independent variable, and age as a covariate, since it could act a confounding factor [61]. Post hoc comparisons were Bonferroni corrected to adjust the level of significance to the multiple comparisons made, and the partial squared Eta (*η_p_*^2^) statistic was used to measure the effect size, with the cut-off points being 0.01 (small), 0.06 (moderate), and 0.14 (large) [62]. Finally, we conducted stepwise linear regressions considering only the significant variables (*p* ≤ 0.05) found in the previous bivariate correlation analysis performed between each TCI-R dimension and the clinical data.

All the data were analyzed using the SPSS software (IBM Corp, Armonk, NY, USA) for Windows, version 25, and tests were two-tailed with the type I error set at 5%. 

## 3. Results

### 3.1. Sociodemographic and Clinical Characteristics 

Table 1 presents the sociodemographic data for the three groups. Mean age for the total sample was 36.95 ± 8.09 years old. Most of the participants were single, lived in company, and were inactive from work; the average years of schooling for the total sample was 9.90 ± 2.23. We found differences in civil status between the SUD and SZ- groups (*p* = 0.013). In the SUD group, a higher proportion of patients were married or had a stable partner. Furthermore, this group presented a higher proportion of working patients than the other two groups (*p* ≤ 0.010 in both cases). 

Regarding the clinical data in Table 2, we did not find differences among the groups in the presence of a family history of mental disorders, but there were differences in the family history of SUD, which were higher in the SZ+ and SUD groups compared to the SZ group (*p* ≤ 0.031 in both cases). No differences were observed in the number of comorbid organic pathologies.

The SZ+ group presented a higher number of suicide attempts than the SUD group (*p* = 0.011), with no differences among the rest of the contrasts, although the SUD group had the lowest rate of previous suicide attempts. Furthermore, the SUD group presented a higher GAF than the two groups with SZ (*p* < 0.001 in both cases). Regarding medication, the SUD group had fewer prescribed drugs compared to the other two groups (*p* < 0.001 in both cases), with no differences between the groups with SZ. When we analyzed the prescription of antipsychotic drugs, no patient in the SUD group had received typical antipsychotics, and only 3.6% of these patients had received atypical antipsychotics. Thus, we found differences in the type of antipsychotic drug prescribed between the SUD group and the two groups with SZ (*p* < 0.001, in all cases), while there were no differences between the SZ+ and SZ- groups. However, when we looked at the doses of antipsychotics converted to milligrams of chlorpromazine (of which the SUD group presented a residual amount), the SZ- group had been prescribed almost twice as many milligrams than the SZ+ group (*p* < 0.001). In the SZ+ group, there were more participants who had been prescribed an interdictory drug compared to the SUD group (*p* = 0.049).

Considering the clinical characteristics of SZ, we found no differences between groups in age of onset or duration of the disorder. We also found no differences in positive or negative symptoms measured with the PANSS, but the SZ+ group had a higher score than the SZ- group on the general psychopathology scale (*p* = 0.004). 

With respect to the clinical characteristics of SUD, we observed that the SZ+ group presented an earlier onset of the disorder with respect to the SUD group (*p* = 0.019), as well as a longer duration of the disorder (*p* = 0.049). Furthermore, the SZ+ group had consumed a greater number of substances on average (*p* = 0.033), but we found no differences in the main type of substance, with a majority of polyconsumers in both groups. Most of the participants had been cocaine and alcohol users, with no differences found between the SUD and SZ+ groups. Neither did we find differences in the consumption of hallucinogens or opioids, although in both cases the rates were higher in the SZ+ group. In contrast, use of cannabis (*p* = 0.010), and of hypnotics and anxiolytics (*p* = 0.008), were higher in the SZ+ group compared to the SUD group. We also found no difference in abstinence time or severity of addiction. Finally, the SZ+ group presented a greater number of previous relapses than the SUD group (*p* = 0.002).

### 3.2. Personality Dimensions

Table 3 shows the results obtained in the TCI-R for the three groups. Regarding the Temperament dimensions, the MANCOVA showed differences among the groups in Novelty Seeking, Harm Avoidance, and Persistence. Thus, the groups with consumption (SZ+ and SUD) presented higher scores in Novelty Seeking, compared to the SZ- group (*p* ≤ 0.001 in both cases). In contrast, the two groups with SZ (SZ+ and SZ-) obtained higher scores in Harm Avoidance (*p* < 0.001 in both cases) and lower Persistence scores (*p* ≤ 0.024 in both cases) with respect to the SUD group.

For the Character dimensions, the MANCOVA contributed differences in Self-directedness and Self-transcendence. Post hoc contrasts showed a lower score for Self-directedness in the SZ+ group compared to the other two groups (*p* < 0.001 in both cases). Finally, the two groups with SZ presented higher scores in Self-transcendence compared to the SUD group (*p* ≤ 0.002 in both cases). 

**Figure 1:**
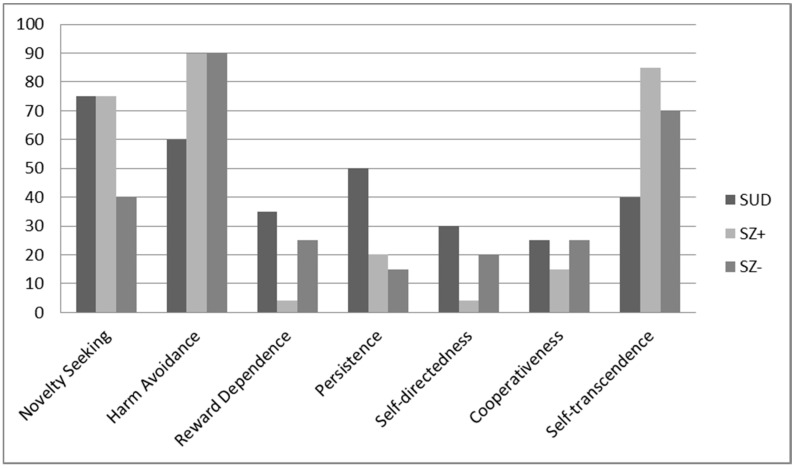
Percentile scores for the three groups for the Temperament and Character Inventory-Revised according to population norms. SUD: Substance use disorder; SZ+: Dual schizophrenia; SZ-: Schizophrenia.

Analysis of the percentiles (Figure 1) showed that the two groups with consumption (SZ+ and SUD) presented high scores in Novelty Seeking, while those of the SZ- group were slightly low in this dimension. On the other hand, the two groups with SZ presented a very high score in Harm Avoidance, while all three groups presented low scores in Reward Dependence, more so in the SZ+ group. Regarding the Persistence scale, the scores for the two groups with SZ were low in this dimension. Regarding the character dimensions, the three groups showed low scores in Self-directedness and Cooperativeness, especially the SZ+ group. Finally, the two groups with SZ presented high scores in Self-transcendence, although more markedly so in the SZ+ group.

### 3.3. Clinical Variates Associated with Personality Dimensions

Table 4 shows the stepwise regression analyses for the TCI-R dimensions. We observed that, for the SUD group, onset age of SUD was negatively related to Novelty Seeking, explaining 13.3% of the variance (F_(1,53)_ = 9.227; *p* = 0.004), while the GAF was positively linked to Reward Dependence, and explained 24.9% of the variance (F_(81,53)_ = 18.885; *p* < 0.001). Regarding the SZ+ group, general psychopathology (PANSS) was positively associated to Harm Avoidance, accounting for 11.7% of the variance (F_(1,53)_ = 6.156; *p* = 0.018). Abstinence period measured in months (*p* = 0.015) was positively related to Self-directedness, whereas general psychopathology (PANSS) (*p* = 0.028) was negatively linked to this dimension, and both of them accounted for 31.3% for the variance (F_(1,53)_ = 9.867; *p* < 0.001) in Self-directedness. Likewise, 28.2% of the variance (F_(1,53)_ = 8.058; *p* < 0.001) in Cooperativeness was explained by number of relapses (*p* = 0.001), which were negatively related, duration of SZ (measured in years) (*p* = 0.005) and onset age of SUD (*p* = 0.036), which were positively related to Cooperativeness in this group. In addition, positive symptoms (PANSS) were positively associated to Self-transcendence and accounted for 8.4% of the variance (F_(1,53)_ = 4.554; *p* = 0.039). Finally, for the SZ- group, positive symptoms (PANSS) were positively related to Harm Avoidance and explained 9.1% of the variance (F_(1,53)_ = 5.096; *p* = 0.019). Similarly, 18.7% of the variance (F_(1,53)_ = 6.627; *p* = 0.003) in Reward Dependence was explained by the GAF (*p* = 0.008) and the PANSS negative symptoms (*p* = 0.032). The former was positively associated to Reward dependence, whereas the latter was negatively associated. General psychopathology was negatively linked both to Self-directedness, and explained 16.1% of the variance (F_(1,53)_ = 10.37; *p* = 0.002), and to Cooperativeness, accounting for 22.8% of the variance in this dimension (F_(1,53)_ = 15.473; *p* < 0.001). General psychopathology was also positively related to Self-transcendence, explaining 11.7% of the variance (F_(1,53)_ = 11.569; *p* = 0.001). 

## 4. Discussion

Our work focused on studying the temperament and character profile of patients with SZ+, SZ- and SUD, comparing them among themselves as well as with respect to the normative reference data, in order to elucidate the existence of a possible endophenotype of SZ+. Furthermore, we tried to study the possible associations among personality traits and the clinical characteristics of each diagnosis.

Regarding the clinical and sociodemographic characteristics, our results are in line with the data provided by previous works analyzing similar groups of patients [14,63,64]. Thus, presenting a SUD, SZ- or SZ+ was associated with being single or without a stable partner, living with the family of origin, having a low educational level, and with being unemployed. Furthermore, the SUD group presented a better sociodemographic and clinical profile compared to the other two groups, thus confirming a greater deterioration associated with SZ compared to SUD [15,25].

The two groups with consumption presented more family history of SUD, which is consistent with the idea that such history is a risk factor for developing an addiction [65]. This reflects the importance of considering this factor in the prevention of addictions. On the other hand, the SZ+ group presented an earlier onset of the disorder, a longer duration of the SUD, and a greater quantity of substances consumed. All of these characteristics have been previously related to a worse clinical state, a greater number of relapses, and more suicide attempts [9,10,15]. In line with previous works [15,27], we observed a higher consumption of cannabis in the SZ+ group, which is of special interest since the evidence points to the consumption of this substance as a risk factor for the development of a SZ [66,67]. Moreover, it becomes even more relevant when we consider that, in our study, the onset of SUD happened earlier than that of SZ in the SZ+ group, in line with that found in previous studies [15,30]. Thus, it is important to pay attention to the consumption of cannabis (especially among people with a higher risk of developing a psychotic disorder), given the high prevalence of its consumption among the youth population [11]. However, in our sample, we have a majority of polyconsumers in both groups, and this does not allow us to analyze these data in greater depth at this time.

Regarding personality results, patients in the groups with consumption (SUD and SZ+) showed a greater tendency to present intense responses to novel stimuli, as well as to respond impulsively and to try to avoid monotony and routine (higher Novelty Seeking), compared with the SZ- group and with population data. This is consistent with the data pointing at Novelty/Sensation Seeking as a possible risk factor for addiction [37,68], regardless of whether or not there is an additional diagnosis of SZ [52]. Furthermore, in the SUD group, a higher Novelty Seeking score was associated with a lower onset age of SUD. This points to the importance of detecting extreme scores for this trait in the population at risk, since an earlier start of consumption has been related to a worse clinical course and higher rates of abandonment of treatment [9,27,44], worse strategies of coping during the therapeutic approach [64], and worse cognitive functioning [69]. Therefore, in interventions with these groups of patients it may be very useful to implement strategies aimed at improving decision making, managing routine or boredom, and directing the search for new sensations towards behaviors different from consumption.

Group members diagnosed with SZ tended to experience negative affect frequently, be pessimistic, have multiple concerns, and some behavioral inhibition (high Harm Avoidance). In line with that observed in other studies, which point to Harm Avoidance as a possible endophenotype for SZ [29,48], patients in our sample with a diagnosis of SZ had higher scores in this dimension than the SUD group or the general population. Furthermore, consistent with previous data [30], higher Harm Avoidance was associated in these groups with the presence of more psychiatric symptoms. This reflects the importance of using treatment interventions aimed at improving the management of negative affect in these patients.

In addition, the three groups were characterized as being less sentimental and more solitary and resistant to social pressure, compared to population data (lower Reward Dependence). This trend was especially marked in the SZ+ group, while the SUD group was more similar to the general population, although there were no differences among the groups in direct scores. These data are consistent with those provided by previous studies, especially regarding the groups with SZ [30,70]. In the SUD and SZ- groups, a closer approach to social stimuli (high Reward Dependence) was associated with better general functioning and, additionally, in the SZ- group it was associated with a lower presence of negative symptoms. This reveals the importance of considering social behavior in order to design therapeutic interventions as well as in studying this dimension further. The two SZ groups showed more difficulties in persisting in a behavior or a task in the face of frustration or fatigue, thus showing a lack of constancy and activity (lower Persistence) compared to the SUD group and the normative data. These results agree with those obtained in previous works regarding groups with SZ [30,70]. This trait (lower Persistence) should also be taken into account, since it may impair treatment involvement and be associated with a higher dropout rate. Thus, at the clinical level, it could be useful to implement motivation and coping strategies to improve therapeutic alliance and adherence to treatment, since the treatment of SZ requires a high degree of compliance for it to be effective, and such treatments are long-lasting given the chronicity of the disorder.

Regarding the Character dimensions, and in line with previous studies [30,44,70], the three groups presented feelings of ineffectiveness as well as difficulties in taking responsibility for their own behavior, for directing it towards their goals and for adapting it to the demands of the situation (low Self-directedness). This trend was especially marked in the groups with SZ where, in addition, low Self-directedness was associated with the presence of more psychiatric symptoms. This reflects the importance of working on self-esteem and coping strategies in these patients, in order to improve not only their adaptive capacity, but also their own psychiatric symptoms.

Moreover, the three groups showed difficulties in adapting to society, a tendency to ignore other people’s needs, and to show little or no interest in social relations (low Cooperativeness) [30,46,48]. This was associated with a greater number of psychiatric symptoms in the SZ group. In addition, in the SZ+ group the duration of the SZ and of the SUD was related to the scores in Cooperativeness, while a greater ease of adapting to social requirements was associated with fewer relapses in this group. Since in the SZ+ group a later SUD onset is associated with more Cooperativeness (feeling comfortable in a group and coping well), future studies may further investigate this trait as a potential protection factor for the onset of a SUD in patients with SZ. Thus, our result emphasizes the importance of paying attention to this personality trait, and of implementing interventions aimed at improving social skills and empathy in the diagnoses considered, in order to prevent relapses and to improve psychiatric symptoms.

Finally, according to previous data [29,30,31], the two SZ groups showed a tendency to spirituality and magical thinking, to have abstract beliefs, and to be carried away by their emotions (higher Self-transcendence), compared to the SUD group and to population data. This trend, which was especially marked in the SZ+ group, was associated in both SZ groups with the presence of more psychiatric symptoms. Thus, a diagnosis of SZ would imply the presence of ideas with a lower reality base, which is consistent with the type of mental disorder in these patients, and which may hinder their treatment. The trait of higher Self-transcendence may thus suppose a certain risk for SZ+ patients in not being realistic in establishing their recovery goals, as well as not being able to detect risk situations during the relapse prevention phase.

Taken as a whole, our results support the idea that there is a character and temperament profile more associated with SUD, and a different one more associated with SZ, and that both differ from the profile reflected by the population data. Thus, patients with SZ would present a specific profile [29,31] characterized by a tendency to negative affect, behavioral inhibition, spirituality and magical thinking, being lonely, with feelings of ineffectiveness and low control of their own behavior, and difficulties in adapting to the social environment. On the other hand, in line with the available data, our results suggest that the SZ+ group presents a personality profile similar to that of the SZ- group, together with the characteristics associated to SUD of low tolerance to routine and monotony, and a tendency to abandon tasks in the face of frustration or fatigue [30,52]. Finally, the SZ+ group presented more marked personality traits than those in the SUD and SZ- groups, in line with what has been observed in previous works [52,53]. This confirms the severity of this diagnostic condition and points to the possible existence of a shared endophenotype for SZ+, made up of traits characteristic of SZ and SUD, and with more extreme values than those observed in these diagnostic entities separately. Furthermore, given that the personality traits are modifiable, the consideration of these behavioral endophenotypes may be of help in designing specific intervention strategies, both for treatment and for relapse prevention.

This study has some strengths and limitations. One of the strengths is having studied these three groups of patients, thus allowing us to overcome the limitation of extrapolating data on patients with SZ+ from patients with SUD, on the one hand, and with SZ- on the other. Furthermore, we consider that the sample is representative of consuming patients, since the main pattern was that of poly-consumption, both with SZ and without psychiatric comorbidity. However, this point may also be a limitation, as it does not allow us to study the differential effects of each substance, nor study if certain personality profiles are associated with the consumption of a specific substance. In this sense, we consider it of special interest to explore further the possible implications of cannabis consumption, which was the largest in the SZ+ group. Moreover, our results are only generalizable to persons with a SUD diagnosis in initial remission, and we have not controlled for the possible effect of time of abstinence. Additionally, the fact that it is a multicenter study confers some external validity to our results. The fact that the sample is made up only of men means that the differences found are not due to sex but, in turn, does not allow us to generalize the results to the female population. This may be a future line of research, despite the greater difficulty of obtaining women undergoing treatment for SUD (around 20%). Furthermore, the study of personality traits has the specific limitations of self-reported measurements. Finally, the cross-sectional and retrospective design does not allow us to establish causal relationships or determine to what extent the results observed in personality traits reflect the effects of treatment. This aspect is of special relevance in the study of personality traits, since, as it has been already commented, several works point to the possibility that these traits may be endophenotypes for SUD and/or SZ. In this sense, we think that it is necessary to carry out longitudinal studies in order to study these aspects in greater depth.

## 5. Conclusions

To the best of our knowledge, this is the first work to study the temperament and character profiles of these three groups of patients, and to relate these dimensions to clinical characteristics of interest. In addition, it allows exploring the specific weight of the characteristics of each of the disorders (SUD and SZ) in SZ+.

The three diagnostic groups presented a different profile from that observed in the general population, with more maladaptive personality characteristics. Our results support the idea of the existence of a personality profile associated with SUD, a different one associated with SZ, and both emerging in combination in the SZ+ group. Thus, the groups with consumption issues presented higher scores in Novelty Seeking, while the groups with SZ presented higher scores in Harm Avoidance and Self-transcendence. In addition, the SZ+ group presented a character and temperament profile with more extreme scores. Regarding the therapeutic approach for these patients, it could be useful to work on their self-esteem, providing specific resources so that they feel capable of taking responsibility for their behavior, implementing strategies to improve their social skills, empathy and collaboration, and working on the interpretation of psychotic symptoms and the content of thoughts. On the other hand, the association of certain personality traits with clinical characteristics (GAF, psychiatric symptoms, onset age of SUD, abstinence period, relapses and duration of SZ) seems to us of special relevance based on the diagnosis, and we consider it a promising line of research, given its clinical applicability. Thus, future studies that overcome the limitations of this work could provide data of great interest in order to design personalized prevention and treatment strategies.

## Figures and Tables

**Table 1:** Sociodemographic data for the three groups. Mean, standard deviation or percentages, and statistical contrasts.

Sociodemographic Variables	SUD (N = 55)	SZ+ (N = 55)	SZ- (N = 55)	Statistical Contrasts
Age	35.78 ± 6.98	36.00 ± 8.19	39.07 ± 8.72	F_(2,162)_ = 2.91
Civil status				χ^2^_(2)_ = 9.75 *
Single	58.2%	76.4%	83.6%	
Married/Stable partner	25.5%	12.7%	9.1%	
Separated/Divorced	16.4%	10.9%	7.3%	
Living arrangements				χ^2^_(1)_ = 2.16
Alone	10.9%	7.3%	3.6%	
Accompanied	89.1%	92.7%	96.4%	
Employment situation				χ^2^_(4)_ = 62.19 ***
Working	30.9%	10.9%	9.1%	
Unemployment compensation	25.5%	5.5%	3.6%	
On sick leave	16.4%	7.3%	0%	
Disability pension	12.7%	61.8%	81.8%	
No income	14.5%	14.5%	5.5%	
Years of schooling	10.38 ± 2.20	9.62 ± 2.31	9.71 ± 2.15	F_(2,162)_ = 1.94

SUD: Substance use disorder; SZ+: Dual schizophrenia; SZ-: Schizophrenia; * *p* < 0.05; *** *p* < 0.001.

**Table 2:** Clinical data for the three groups. Mean, standard deviation or percentages, and statistical contrasts.

Clinical Characteristics	SUD (N = 55)	SZ+ (N = 55)	SZ- (N = 55)	Statistical Contrasts
Family history of psychiatric disorders	21.8%	29.1%	34.5%	χ^2^_(1)_ = 2.20
Family history of SUD	29.1%	21.8%	7.3%	χ^2^_(1)_ = 8.68 *
Suicide attempts	0.42 ± 0.90	1.25 ± 1.82	0.69 ± 1.57	F_(2,162)_ = 4.56 *
GAF	74.50 ± 10.06	63.13 ± 11.22	59.75 ± 10.15	F_(2,162)_ = 29.52 ***
Number of psychiatric medications	0.93 ± 1.14	3.30 ± 1.68	3.22 ± 1.46	F_(2,162)_ = 47.81 ***
Typical antipsychotics	0%	22.2%	25.5%	χ^2^_(1)_ = 15.80 ***
Atypical antipsychotics	3.6%	96.3%	94.5%	χ^2^_(1)_ = 134.74 ***
CPZ equivalent dosage (mg)	6.06 ± 32.13	350.55 ± 281.35	617.07 ± 522.12	F_(2,162)_ = 43.56 ***
Interdictor	20%	37%		χ^2^_(1)_ = 3.89 *
Medical disease comorbidity	0.47 ± 0.69	0.53 ± 0.77	0.64 ± 0.80	F_(2,162)_ = 0.67
Onset age of SZ		23.35 ± 6.96	23.65 ± 6.71	t_(1,108)_ = 0.237
Duration of SZ (years)		12.65 ± 8.01	15.42 ± 9.30	t_(1,108)_ = 1.67
PANSS scores				
Positive symptoms		11.83 ± 5.70	10.30 ± 4.19	t_(1,108)_ = 1.46
Negative symptoms		15.58 ± 7.39	14.18 ± 7.40	t_(1,108)_ = 0.89
General psychopathology		31.10 ± 10.91	24.70 ± 9.01	t_(1,108)_ = 2.99 **
Onset age of SUD	20.55 ± 7.24	17.60 ± 5.65		t_(1,108)_ = 2.38 *
Duration of SUD (years)	14.61 ± 8.94	17.85 ± 8.03		t_(1,108)_ = 2.00 *
Number of substances used	2.93 ± 1.61	3.62 ± 1.75		t_(1,108)_ = 2.16 *
Main substance of dependence				χ^2^_(4)_ = 6.66
Cocaine	12.7%	10.9%		
Alcohol	9.1%	12.7%		
Alcohol + Cocaine	27.3%	9.1%		
Polydrug use	50.9%	67.3%		
Type of substances used ^a^				
Cocaine	89.10%	92.70%		χ^2^_(1)_ = 0.44
Alcohol	80.00%	76.0%		χ^2^_(1)_ = 0.21
Cannabis	52.70%	76.40%		χ^2^_(1)_ = 6.71 **
Psychodysleptics	27.30%	40.00%		χ^2^_(1)_ = 1.99
Opioids	14.50%	25.50%		χ^2^_(1)_ = 2.05
Sedatives	1.80%	16.40%		χ^2^_(1)_ = 7.04 **
Abstinence period (months)	7.55 ± 2.61	6.57 ± 3.64		t_(1,108)_ = 1.62
Number of relapses	0.82 ± 1.48	2.25 ± 2.97		t_(1,108)_ = 3.21 **
DAST-20 (severity of addiction)	13.05 ± 3.47	13.44 ± 2.86		t_(1,108)_ = 0.54

SUD: Substance use disorder; SZ+: Dual schizophrenia; SZ-: Schizophrenia; GAF: Global Assessment of Functioning; CPZ: Chlorpromazine; SZ: Schizophrenia; PANSS: Positive and Negative Syndrome Scale; DAST-20: Drug Abuse Screening Test; * *p* < 0.05; ** *p* < 0.01; *** *p* < 0.001; ^a^ Percentages will not equal 100 as each patient may have taken more than one substance.

**Table 3:** Results for the Temperament and Character Inventory-Revised (TCI-R) dimensions for the three groups. Mean, standard error, and MANCOVA results.

TCI-R Dimensions	SUD (N = 55)	SZ+ (N = 55)	SZ- (N = 55)	F_(2,161)_	*η_p_* ^2^	Bonferroni *Post-Hoc* Analyses
**Temperament**						
Novelty Seeking	106.88 ± 1.86	106.06 ± 1.86	96.23 ± 1.88	9.86 ***	0.11	SUD,SZ+ > SZ-
Harm Avoidance	95.27 ± 2.60	112.12 ± 2.60	111.18 ± 2.62	13.22 ***	0.14	SZ+,SZ- > SUD
Reward Dependence	97.88 ± 2.14	91.50 ± 2.14	95.70 ± 2.16	2.31	0.03	
Persistence	113.48 ± 2.66	103.45 ± 2.65	99.38 ± 2.68	7.38 ***	0.08	SZ+,SZ -< SUD
**Character**						
Self-directedness	137.82 ± 3.08	116.54 ± 3.08	134.64 ± 3.11	13.90 ***	0.15	SZ+ < SZ-,SUD
Cooperativeness	130.75 ± 2.44	123.24 ± 2.43	131.40 ± 2.46	3.46	0.04	
Self-transcendence	58.66 ± 2.20	77.26 ± 2.20	69.77 ± 2.22	18.19 ***	0.18	SZ+,SZ- > SUD

SUD: Substance use disorder; SZ+: Dual schizophrenia; SZ-: Schizophrenia; *** *p* < 0.001.

**Table 4:** Multiple linear regression models for the Temperament and Character Inventory-Revised (TCI-R) for the three groups.

TCI-R Dimensions	SUD (N = 55)			SZ+ (N = 55)			SZ- (N = 55)		
	R^2^ Adjusted	IV	β Standardized	R^2^ Adjusted	IV	β Standardized	R^2^ Adjusted	IV	β Standardized
**Temperament**									
Novelty Seeking	0.133	Onset age of SUD	−0.386 **						
Harm Avoidance				0.117	PANSS_GP	0.373 *	0.091	PANSS_P	0.331*
Reward Dependence	0.249	GAF	0.513 ***				0.187	GAFPANSS_N	0.360 ** −0.285 *

**Character**									
Self-directedness				0.313	Abstinence period (months)	0.370 * −0.332 *	0.161	PANSS_GP	−0.421 **
					PANSS_GP				
Cooperativeness				0.282	Number of relapses	−0.412 *** 0.343 ** 0.249 *	0.228	PANSS_GP	−0.494 ***
					Duration of SZ (years)				
					Age of SUD onset				
Self-transcendence				0.084	PANSS_P	0.327 *	0.117	PANSS_GP	0.441 ***

SUD: Substance use disorder; SZ+: Dual schizophrenia; SZ-: Schizophrenia; GAF: Global Assessment of Functioning; SZ: Schizophrenia; PANSS_GP: Positive and Negative Syndrome Scale_General Psychopathology; PANSS_P: Positive and Negative Syndrome Scale_Positive Scale; PANSS_N: Positive and Negative Syndrome Scale_Negative Scale; * *p* < 0.05; ** *p* < 0.01; *** *p* < 0.001.
